# Sensitivity analysis for calibrated inverse probability-of-censoring weighted estimators under non-ignorable dropout

**DOI:** 10.1177/09622802221090763

**Published:** 2022-04-12

**Authors:** Li Su, Shaun R Seaman, Sean Yiu

**Affiliations:** MRC Biostatistics Unit, School of Clinical Medicine, 12204 University of Cambridge, UK

**Keywords:** Covariate balancing, informative dropout, inverse probability weighting, longitudinal data, missing not at random

## Abstract

Inverse probability of censoring weighting is a popular approach to handling dropout in longitudinal studies. However, inverse probability-of-censoring weighted estimators (IPCWEs) can be inefficient and unstable if the weights are estimated by maximum likelihood. To alleviate these problems, calibrated IPCWEs have been proposed, which use calibrated weights that directly optimize covariate balance in finite samples rather than the weights from maximum likelihood. However, the existing calibrated IPCWEs are all based on the unverifiable assumption of sequential ignorability and sensitivity analysis strategies under non-ignorable dropout are lacking. In this paper, we fill this gap by developing an approach to sensitivity analysis for calibrated IPCWEs under non-ignorable dropout. A simple technique is proposed to speed up the computation of bootstrap and jackknife confidence intervals and thus facilitate sensitivity analyses. We evaluate the finite-sample performance of the proposed methods using simulations and apply our methods to data from an international inception cohort study of systemic lupus erythematosus. An R Markdown tutorial to demonstrate the implementation of the proposed methods is provided.

## 1 Introduction

### 1.1 Inverse probability-of-censoring weighting and covariate balancing weights

Inverse probability-of-censoring weighting (IPCW) is a popular approach to handling the dropout that is ubiquitous in longitudinal studies.^1,2^ Under the assumption of sequential ignorability, inverse probability-of-censoring weights are usually obtained by specifying a parametric model for the dropout process and estimating its parameters using maximum likelihood estimation (MLE). However, it is well known that inverse probability-of-censoring weighted estimators (IPCWEs) using weights estimated by MLE can be inefficient and unstable, especially when the dropout model is misspecified.^3–5^

Covariate balancing weight (CBW) methods have been proposed as a way of improving the performance of inverse probability weighted estimators in the setting where the aim is to estimate the causal effect of a binary point treatment (e.g.^6–10^). Empirical and theoretical studies have shown that CBW methods reduce the mean squared errors (MSEs) of the inverse probability weighted estimators under both correct and incorrect model specifications.^11^ Recently, CBW methods have been developed to improve IPCWEs. Han^12^ proposes a calibration approach for IPCWEs when the aim is to estimate the mean of an outcome measured at the end of a longitudinal study. The calibration restrictions proposed by him aim to balance, at each follow-up visit, the predicted outcome from each of a set of models for the expected outcome given the history of a set of time-varying covariates. If one of the models for predicting the outcome is correctly specified, the IPCWE using calibrated weights is consistent. To improve the inverse probability weighted estimators in marginal structural models,^1^ Yiu and Su proposed to jointly calibrate inverse probability of treatment and censoring weights.^13^ They extended the ‘covariate association eliminating weights’ method in Yiu and Su^14^ to the ignorable dropout setting and provided a coherent framework for deriving calibration restrictions in longitudinal studies. Unlike the approach of Han^12^, that of Yiu and Su^13^ can handle repeatedly measured outcomes.

A limitation of the existing calibrated IPCWEs is that they are based on the *unverifiable* assumption of sequential ignorability, that is, the assumption that the probability of dropout between any two consecutive visits (say visit j−1 and visit j ) is independent of future outcomes given the data that have been observed up to visit j−1 . In practice, it is desirable to assess the sensitivity of conclusions from the IPCWEs to violations of this sequential ignorability assumption. For IPCWEs without calibration, sensitivity analysis approaches under non-ignorable dropout have been developed. For example, Rotnitzky et al.^15^ described how to fit a marginal model for a repeatedly measured outcome in a longitudinal study when the probability of dropout between visits j−1 and j may depend on the potentially unobserved outcome at visit j (and possibly also the outcomes at later visits). Their method involves specifying a ‘selection function’, which characterizes the residual dependence of the probability of dropout between visits j−1 and j on the outcome at visit j (and possibly later visits) after conditioning on the observed covariates and outcomes up to visit j−1 . This selection function involves a sensitivity parameter (or a vector of parameters) that describes the strength of the residual dependence. By varying this sensitivity parameter over a range of plausible values, the sensitivity of the substantive conclusions to deviations from sequential ignorability can be assessed. Other examples of methods using such selection functions include Scharfstein et al., Vansteelandt et al., and Wen and Seaman.^16–18^

To address the aforementioned limitation, in this paper, we propose a sensitivity analysis approach for calibrated IPCWEs under non-ignorable dropout. Building upon the calibration approach of Yiu and Su,^13^ we incorporate a selection function with sensitivity parameters into the model used for estimating an initial set of inverse probability-of-censoring weights before calibration. Then we calibrate these initial weights by balancing the distributions of observed covariates after weighting with the observed covariate distributions in the target population in the absence of dropout. Although confidence intervals (CIs) for our calibrated IPCWEs can be based on nonparametric bootstrap (or jackknife if the sample size is small), it is time-consuming to repeat the whole process for each value of the sensitivity parameter(s) and each bootstrap sample. We therefore propose a simple technique to speed up the computation of the bootstrap/jackknife CIs and evaluate its performance using simulations.

### 1.2 Motivating example: Cerebrovascular events and health-related quality of life in patients with systemic lupus erythematosus

This work was motivated by a study of the impact of cerebrovascular events (CerVEs) on health-related quality of life (HRQoL) in patients with systemic lupus erythematosus (SLE).^19^ Neuropsychiatric (NP) events are frequent in patients with SLE, a chronic autoimmune disease that affects multiple organ systems. CerVEs (e.g. stroke and transient ischaemia) are the fourth most frequent NP event in SLE and are usually attributable to SLE. Regardless of attribution, NP events are associated with a negative impact on HRQoL in both cross-sectional and longitudinal studies. In a recent study conducted by the Systemic Lupus International Collaborating Clinics (SLICC) group, it was found that CerVEs were associated with a significant and sustained reduction in patient self-reported HRQoL even after adjusting for other factors predicting HRQoL.^19^ This finding was based on data from the SLICC inception cohort, where 1826 SLE patients were enrolled within 15 months of their SLE diagnosis between October 1999 and December 2011 and followed up annually thereafter at 36 academic medical centres from 12 countries.

The analysis reported in Hanly et al.^19^ was based on the generalized estimating equation (GEE) approach with the AR(1) working correlation structure, and the variation in the length of follow-up in the SLICC cohort was not taken into account. In fact, by the study cut-off date of 10 December 2015, the number of annual assessments per patient varied from 1 to 18; the mean follow-up time was 6.6 years and the standard deviation was 4.1 years. Specifically, 46% of the SLICC patients had their latest annual assessment visit more than 2 years before the study cut-off date. These patients may have dropped out of the study. Since the current HRQoL of a patient is likely to be associated with his/her probability of dropping out of the study, there could be non-ignorable missingness in the longitudinal HRQoL data of the SLICC cohort. This motivates us to re-analyze the SLICC HRQoL data, taking into account possibly non-ignorable dropout in this cohort.

## 2 Methods

### 2.1 Notation, setting and assumptions

We consider a study in which n independent patients are enrolled at baseline (denoted by visit 0 ) and then followed up over time at scheduled visits 1,…,T . Note that in Section 4.2 we shall extend our proposed method to allow individuals to have different maximum numbers of follow-up visits (i.e. $T=T_{i}$ ), for example, due to administrative censoring as a result of staggered entry to the SLICC cohort. We also assume that the visiting times are non-informative, that is, scheduled by the study design rather than initiated by patients. Note that GEE-based methods in general (including the proposed method) can be biased when the visiting times depend on the outcomes (see Pullenayegum and Lin^20^).

Let $V_{i}$ denote baseline covariates (e.g. demographics) recorded for the i th patient at enrolment. Let $X_{ij}$ and $Y_{ij}$ denote the values of, respectively, a p×1 vector of time-varying covariates and a longitudinal outcome for the i th patient at his/her scheduled j th visit (visit j , j=0,1,…,T ). Let $R_{ij}$ be the indicator of whether the i th patient remains in the study at the time of visit j . Thus when $R_{ij}=0$ , $Y_{ij}$ is missing and $X_{ij}$ could be missing. We assume that $R_{i0}=1$ (i.e. baseline visit assessments are complete for all patients) and $R_{i,j-1}=0\Rightarrow R_{ij}=0$ (monotone missingness due to dropout).

For simplicity, in this paper, we focus on linear models for $E(Y_{ij}|X_{ij})$ , but the proposed method can be straightforwardly applied to generalized linear models.^21^ Specifically, our interest is in estimating the parameters of an outcome model

(1) $$E(Y_{ij}|X_{ij})=\mu_{ij}=X_{ij}^{\text{T}}\beta ,\;j=0,\ldots ,T$$

where β is a p×1 vector of regression parameters. To achieve this, we make the ‘non-future dependence’ assumption for the dropout process, that is, $\mathrm{pr}(R_{ij}=1|Y_{ij},\ldots ,Y_{iT},H_{i,j-1},R_{i,j-1}=1)=\mathrm{pr}(R_{ij}=1|Y_{ij},H_{i,j-1},R_{i,j-1}=1)$ for j=1,…,T , where $H_{i,j-1}$ includes baseline covariates $V_{i}$ , previous outcome history $Y_{i0},\ldots ,Y_{i,j-1}$ , and time-varying covariates $X_{i0},\ldots ,X_{iT}$ . $H_{i,j-1}$ can also include other prognostic variables of the outcome that are measured before visit j and could have influenced the dropout process. In addition, we make the positivity assumption that if $f(Y_{ij},H_{i,j-1},R_{i,j-1}=1)>0$ then $\mathrm{pr}(R_{ij}=1|Y_{ij},H_{i,j-1},R_{i,j-1}=1)>0$ .

### 2.2 Inverse probability-of-censoring weighting under non-ignorable dropout

The first step in our method is to specify the model that will be used to estimate an initial set of inverse probability-of-censoring weights under non-ignorable dropout. In Section 2.3, we shall describe how these initial weights are calibrated.

Let $\mathrm{pr}(R_{ij}=1|Y_{ij},H_{i,j-1},R_{i,j-1}=1)=\pi_{j}(Y_{ij},H_{i,j-1};\alpha ,\gamma )$ , j=1,…,T and assume that

(2) $$\mathrm{logit}\{\pi_{j}(Y_{ij},H_{i,j-1};\alpha ,\gamma )\}=h(H_{i,j-1};\alpha )+q(H_{i,j-1},Y_{ij};\gamma )$$

where $q(H_{i,j-1},Y_{ij};\gamma )$ is a known selection function with a known sensitivity parameter vector γ , and $h(H_{i,j-1};\alpha )$ is a function of $H_{i,j-1}$ with unknown parameter α . Note that $h(H_{i,j-1};\alpha )$ can only depend on $X_{ij},\ldots ,X_{iT}$ if they are fully observed (i.e. they continue to be observed after dropout). In the analysis of the SLICC cohort data reported in Section 4, we assume that

(3) $$\mathrm{logit}\{\pi_{j}(Y_{ij},H_{i,j-1};\alpha ,\gamma )\}=\alpha_{0}+V_{i}^{\text{T}}\alpha_{v}+X_{i,j-1}^{\text{T}}\alpha_{x}+\alpha_{y}Y_{i,j-1}+\gamma Y_{ij}$$

Here γ characterizes the residual dependence of the probability of observing $Y_{ij}$ on the value of $Y_{ij}$ after adjusting for the observed data up to visit j−1 . $X_{i,j-1}$ includes j−1 and $(j-1{)}^{2}$ , and so the probability of dropout on the logit scale is a quadratic function of the visit number. $\alpha =(\alpha_{0},\alpha_{v},\alpha_{x},\alpha_{y})$ is the parameter vector to be estimated.

If $q(H_{i,j-1},Y_{ij};\gamma )=0$ , then the dropout process is sequentially ignorable. Standard logistic regression can then be used to estimate α . However, when $q(H_{i,j-1},Y_{ij};\gamma )\neq 0$ , standard logistic regression cannot be applied, because it will involve the missing $Y_{ij}$ . Following Wen and Seaman,^18^ for a fixed value of γ , we estimate α by solving the following estimating equations

(4) $$\sum_{i=1}^{n}\sum_{\;j=1}^{T}\phi (H_{i,j-1})R_{i,j-1}\left\{\frac{R_{ij}}{\pi_{j}(Y_{ij},H_{i,j-1};\alpha ,\gamma )}-1\right\}=0$$

where $\phi (H_{i,j-1})$ is a vector of functionals of $H_{i,j-1}$ (including 1) and has the same dimension as α . Equation (4) are unbiased estimating equations because $\mathrm{pr}(R_{ij}=1|Y_{ij},H_{i,j-1},R_{i,j-1}=1)=\pi_{j}(Y_{ij},H_{i,j-1};\alpha ,\gamma )$ implies that the expectation of $\left\{\frac{R_{ij}}{\pi_{j}(Y_{ij},H_{i,j-1};\alpha ,\gamma )}-1\right\}$ given $R_{i,j-1}=1$ is zero.

In the SLICC data example, we use $\phi (H_{i,j-1})=(1,V_{i}^{\text{T}},X_{i,j-1}^{\text{T}},Y_{i,j-1})$ . Note that when $R_{ij}=0$ , $R_{ij}/\pi_{j}(Y_{ij},H_{i,j-1};\alpha ,\gamma )=0$ for all values of $Y_{ij}$ and $H_{i,j-1}$ (given the positivity assumption). Thus solving (4) does not involve the missing value of $Y_{ij}$ .

The Newton-Raphson algorithm can be applied to solve (4). Let $\hat{\alpha }$ be the estimator of α obtained by solving (4). Wen and Seaman^18^ proved that $\hat{\alpha }$ is consistent if the dropout model (2) is correctly specified and the selection function, including the sensitivity parameter, is correctly chosen. Given $\hat{\alpha }$ and γ , the inverse probability-of-censoring weights can be obtained by calculating

$$W_{ij}(\hat{\alpha },\gamma )=\prod_{k=1}^{\;j}1/\pi_{j}(Y_{ik},H_{i,k-1};\hat{\alpha },\gamma )$$

For convenience, we call these weights the ‘MLE weights’, even though (4) are not the score equations for α . With the MLE weights, the following estimating equations can be used to consistently estimate β ,

(5) $$\sum_{i=1}^{n}U_{i}(\beta )=\sum_{i=1}^{n}\sum_{\;j=0}^{T}\left(\frac{\partial \mu_{ij}}{\partial \beta }\right)W_{ij}(\hat{\alpha },\gamma )R_{ij}(Y_{ij}-\mu_{ij})=0$$

where at baseline visits $W_{i0}(\hat{\alpha },\gamma )=1$ . In this paper, we use the MLE weights as the initial weights.

### 2.3 Calibrating inverse probability-of-censoring weights under non-ignorable dropout

The purpose of IPCW is to create a representative sample of the target population (i.e. the study population at baseline) in the absence of dropout. As an alternative to the MLE approach, the calibration approach^12,13^ aims to create a representative sample of the target population by balancing the distributions of observed covariates after weighting with those in the target population. Specifically, weights are obtained by calibrating/adjusting the initial set of inverse probability-of-censoring weights (i.e. the MLE weights) such that moment conditions of observed covariates (i.e. calibration restrictions) are satisfied in the finite sample.

#### 2.3.1 Calibration restrictions

Let $W_{ij}^{C}(\lambda )$ denote the calibrated weights, where λ is a r×1 parameter vector to be estimated. Note that we use the superscript C to indicate that the weights are calibrated. Following Yiu and Su,^13^ we use the following calibration restrictions

(6) $$\sum_{\;j=1}^{T}(T-j+1)\sum_{i=1}^{n}\left\{R_{ij}W_{ij}^{C}(\lambda )-R_{i,j-1}W_{i,j-1}^{C}(\lambda )\right\}{\tilde{H}}_{i,j-1}=0$$

where ${\tilde{H}}_{i,j-1}$ is a r×1 vector of functionals of $H_{i,j-1}$ including 1 . The term $\sum_{i=1}^{n}\{R_{ij}W_{ij}^{C}(\lambda )-R_{i,j-1}W_{i,j-1}^{C}(\lambda )\}{\tilde{H}}_{i,j-1}$ in (6) can be interpreted as the covariate balance summary of ${\tilde{H}}_{i,j-1}$ between the weighted uncensored observations at visit j and the weighted uncensored observations at visit j−1 .

The restrictions in (6) are equivalent to

(7) $$\sum_{i=1}^{n}\sum_{\;j=1}^{T}R_{ij}W_{ij}^{C}(\lambda )\left\{(T-j+1){\tilde{H}}_{i,j-1}-(T-j){\tilde{H}}_{ij}\right\}=\sum_{i=1}^{n}T{\tilde{H}}_{i0}$$

Since ${\tilde{H}}_{i,j-1}$ ( j=1,…,T ) includes 1, (7) implies $\sum_{i=1}^{n}\sum_{\;j=1}^{T}R_{ij}W_{ij}^{C}(\lambda )=nT,$ which means that the total number of follow-up ‘observations’ after weighting is equal to nT , the total number of follow-up observations there would have been if no dropout had occurred. If ${\tilde{H}}_{i,j-1}$ includes baseline covariates $V_{i}$ , (7) implies $\sum_{i=1}^{n}\sum_{\;j=1}^{T}R_{ij}W_{ij}^{C}(\lambda )V_{i}=\sum_{i=1}^{n}TV_{i},$ that is, the weighted average of $V_{i}$ over all follow-up visits for all patients is equal to the sample average of $V_{i}$ . If ${\tilde{H}}_{i,j-1}$ includes an indicator for visit, I(j=k) ( k=1,…,T ), and an interaction between this visit indicator and $V_{i}$ , that is, $I(j=k)V_{i}$ , then (7) implies $\sum_{i=1}^{n}R_{ik}W_{ik}^{C}(\lambda )=n$ and $\sum_{i=1}^{n}R_{ik}W_{ik}^{C}(\lambda )V_{i}=\sum_{i=1}^{n}V_{i}$ for k=1,…,T . That is, at each follow-up visit, the sample size after weighting is n and the weighted average of $V_{i}$ is equal to the sample average of $V_{i}$ . If interactions between visit indicators and time-varying covariates are included in ${\tilde{H}}_{i,j-1}$ , then the time-varying covariates will be balanced separately at each follow-up visit.

Imposing calibration restrictions of baseline and time-varying covariates separately at each follow-up visit will ensure that covariate distributions are exactly balanced such that a representative sample of the target population is created at each follow-up visit. This is important since the parameters of interest are the regression coefficients of time-varying covariates in the outcome model (1). However, the number of restrictions will then be proportional to the number of follow-up visits, which will result in a large number of restrictions if there are many baseline and time-varying covariates. In this case, we recommend including the interactions between j (treated as a continuous variable instead of binary indicators) and the covariates in ${\tilde{H}}_{i,j-1}$ to provide some parsimony in the calibration restrictions.

In this paper, we consider calibrated weights of the form

$$W_{ij}^{C}(\lambda )=W_{ij}(\hat{\alpha },\gamma )\exp\;\left[\lambda^{\text{T}}\left\{(T-j+1){\tilde{H}}_{i,j-1}-(T-j){\tilde{H}}_{ij}\right\}\right]$$

Although other forms of calibrated weights are possible (e.g. see Han^12^), this particular choice is appealing because solving (7) is then equivalent to minimizing a convex function of λ ^13^; see details in Section 2.3.2.

It is easy to see that if we replace $W_{ij}^{C}(\lambda )$ with the true inverse probability-of-censoring weights $W_{ij}^{*}=1/\prod_{k=1}^{\;j}\mathrm{pr}(R_{ik}=1|Y_{ik},H_{i,k-1},R_{i,k-1}=1)$ , then the population version of (7)

(8) $$\;E\left[\sum_{\;j=1}^{T}R_{ij}W_{ij}^{*}\left\{(T-j+1){\tilde{H}}_{i,j-1}-(T-j){\tilde{H}}_{ij}\right\}\right]=TE({\tilde{H}}_{i0})$$

is satisfied. Therefore, as long as the model (2) for estimating the initial set of weights is correctly specified, the calibrated weights will converge to the true weights, and thus replacing the MLE weights in (5) with the calibrated weights will not affect the consistency of the IPCWE obtained.

#### 2.3.2 Implementation

The calibrated weights $W_{ij}^{C}(\lambda )$ are calculated by finding the value of λ that solves (7), or equivalently by minimizing

(9) $$\sum_{i=1}^{n}\sum_{\;j=1}^{T}R_{ij}W_{ij}^{C}(\lambda )-\sum_{i=1}^{n}T\lambda^{\text{T}}{\tilde{H}}_{i0}$$

Note that the function in (9) is convex in λ , which ensures that the solution to (7) is unique and can be found efficiently. We solve (7) directly by using the R package nleqslv.^22^ For the SLICC data example reported in Section 4, it took less than 1 second to obtain the calibrated weights with 25 calibration restrictions on a Linux machine with a 3.80  GHz CPU and 64 GB memory.

### 2.4 Confidence intervals

#### 2.4.1 Bootstrap and jackknife confidence intervals

Whether using the MLE weights or calibrated weights, CIs can be obtained by nonparametric bootstrap. Specifically, B bootstrap samples are generated by resampling patients in the observed data with replacement. For each bootstrap sample, the MLE weights and calibrated weights are estimated following the methods described in Sections 2.2 and 2.3. ${\hat{\beta }}_{b}$ ( b=1,…,B ) is calculated by solving the estimating equation $\sum_{i=1}^{n}U_{i}(\;\beta_{b})=0$ for the b th bootstrap sample. The bootstrap CIs are then constructed by applying the percentile method to ${\hat{\beta }}_{b}$ ( b=1,…,B ).^23^

For small samples, jackknife is an alternative to bootstrap for obtaining the variance of the IPCWE and constructing CIs. In particular, jackknife can be useful when there are convergence issues for estimating the MLE weights because the Newton–Raphson algorithm for solving (4) breaks down due to ill-conditioned matrices in a particular bootstrap sample. Specifically, we will leave out the i th patient’s data in the i th jackknife sample. The weight estimation and estimation of β are then repeated for the i th jackknife sample. Let ${\hat{\beta }}_{k,i}^{J}$ ( i=1,…,n ) denote the i th jackknife estimate of the k th element of β . We calculate the jackknife standard error of the k th element of β as

$$\frac{1}{n(n-1)}\sum_{i=1}^{n}({\hat{\beta }}_{k,i}^{\;J}-{\bar{\beta }}_{k}^{\;J}{)}^{2}$$

where ${\bar{\beta }}_{k}^{\;J}=\sum_{i=1}^{n}{\hat{\beta }}_{k,i}^{\;J}/n$ . 95% Wald CIs are then constructed using the jackknife standard errors.

The procedures for constructing bootstrap and jackknife CIs are straightforward. However, because the MLE weights need to be estimated and then calibrated for each bootstrap/jackknife sample, repeating this whole process is time-consuming. On top of this, in sensitivity analyses, we need to vary the values of the sensitivity parameter γ and repeat the estimation, calibration and bootstrapping for each fixed value of γ . Therefore, it is desirable to speed up the computation for constructing bootstrap/jackknife CIs when using calibrated weights.

In this paper, we propose to use the MLE weights estimated from the *original data* as the initial weights and then to implement the calibration using the restrictions based on the bootstrap/jackknife sample. This avoids calculating the MLE weights for every bootstrap/jackknife sample. Optimizing covariate balance in finite samples helps to eliminate chance imbalances^24,25^ and thus reduce the estimation error and variance of inverse probability weighted estimators of treatment effects, as shown in many empirical studies.^7,8,14^ Similarly, calibration by covariate balancing can improve the efficiency of the IPCWEs.^9,12^ Since calibration will eliminate chance imbalances in bootstrap/jackknife samples regardless of the initial weights, fixing the initial weights at those estimated from the original data should have minimal impact on the variance and bootstrap/jackknife CIs. In the next section, we will conduct simulation studies to investigate whether re-estimating the initial weights affects the performance of the bootstrap and jackknife CIs with calibrated weights.

#### 2.4.2 Confidence intervals based on sandwich variance estimators

CIs could also be obtained by using a sandwich estimator of the variance of the regression parameters given the estimated weights, that is

$${\left\{\sum_{i=1}^{n}\frac{\partial U_{i}(\hat{\beta })}{\partial \beta^{\text{T}}}\right\}}^{-1}\left\{\sum_{i=1}^{n}U_{i}(\hat{\beta })U_{i}(\hat{\beta }{)}^{\text{T}}\right\}{\left\{\sum_{i=1}^{n}\frac{\partial U_{i}(\hat{\beta })}{\partial \beta^{\text{T}}}\right\}}^{-1}$$

where $\hat{\beta }$ are estimates of β by applying the MLE weights or calibrated weights in (5). However, since the uncertainty of the estimated weights is not accounted for, it is expected that such CIs will be conservative, compared to those based on non-parametric bootstrap. This is because true asymptotic variances are actually greater when true weights are used than when they are estimated. Therefore, ignoring uncertainty in calibrated weights causes over-estimation of variances.^2^ For IPCWEs without calibration, Rotnitzky et al.^15^ provided an alternative sandwich variance estimator, which accommodates the uncertainty due to estimating the parameters in the dropout model. For IPCWEs with calibration, it would be quite complicated to incorporate the uncertainty in the calibrated weights into sandwich variance estimators, because this uncertainty comes from both the estimation of the initial weights and the calibration. This warrants further research.

## 3 Simulation

In this section, we conduct two simulation studies to assess the performance of the proposed methods in finite samples. In the first simulation study, we assess the performance of the IPCWEs using calibrated weights when dropout is non-ignorable and compare this with the performance of IPCWEs using the MLE weights. In the second simulation study, we evaluate the performance of 95% CIs calculated using non-parametric bootstrap, jackknife or the sandwich variance estimators, when either the MLE weights or calibrated weights are used.

### 3.1 Data generating mechanism

The design of the simulation studies is adapted from the simulation settings in Kang and Schafer.^3^ The data generating mechanism for a patient is summarized in Table 1. Note that T=8 . We omit the subscript i for patients for clearer presentation. Our aim is to estimate the regression parameters $\beta_{0}$ and $\beta_{1}$ for the mean of the longitudinal outcome $E(Y_{j})=\beta_{0}+\beta_{1}\;j$ ( j=0,…,8 ). The true values of $\beta_{0}$ and $\beta_{1}$ are 210 and −2 , respectively.

**Table 1.** Data generating mechanism for the simulations.

**Baseline** ( j=0 ) and **follow-up visits** ( j=1,…,8 , T=8 )	
*Baseline covariates*:	V1,V2,V3,V4i.i.d∼N(0,1)
*Random effect*:	Ui.i.d∼N(0,100)
*Outcome*:	$Y_{j}=210-2j+27.4V_{1}+13.7V_{2}+13.7V_{3}+13.7V_{4}+U+\epsilon_{\;j}$ $\epsilon_{\;j}∼N(0,100)$
*Dropout*:	$R_{0}=1$ $R_{j}|R_{\;j-1}=1∼\text{Bernoulli}(\pi_{j})$ $\mathrm{logit}(\pi_{j})=2-V_{1}+0.5V_{2}-0.25V_{3}-0.1V_{4}+0.2Y_{\;j-1}^{*}+\gamma Y_{\;j}^{*}$ , where $Y^{*}=(Y-210)/35$ . *Sequentially ignorable:* γ=0 *Sequentially non-ignorable:* γ=1
*Transformed covariates*:	$Z_{1}=\exp\;(V_{1}/2)$ $Z_{2}=V_{2}/\{1+\exp\;(V_{1})\}+10$ $Z_{3}=(V_{1}V_{3}/25+0.6{)}^{3}$ $Z_{4}=(V_{2}+V_{4}+20{)}^{2}$

In this set-up, there are four baseline covariates $V=\{V_{1},V_{2},V_{3},V_{4}\}$ , which affect the probability of dropping out of the study and $E(Y_{j})$ . In addition, the previous outcome $Y_{\;j-1}$ and the current outcome $Y_{j}$ at visit j could affect the discrete-time hazard of dropout between visits j−1 and j . The dependence on $Y_{j}$ is characterized by the parameter γ . If γ=0 , the dropout process is sequentially ignorable. Correlation between the observations of the longitudinal outcome is induced by a patient-level random effect U . Data from each patient are generated independently. We simulate 2000 data sets with different sample sizes ( n=200,500,1000,2000 ). Approximately 38% and 43% of the data are missing when dropout is sequentially ignorable ( γ=0 ) and non-ignorable ( γ=1 ), respectively.

### 3.2 Performance of the IPCWEs under non-ignorable dropout

#### 3.2.1 Setup

In this section, we evaluate the performance of the IPCWEs using the MLE weights and calibrated weights under both correct and incorrect model specifications when the dropout process is *non-ignorable* (i.e. γ≠0 ). We consider two types of model misspecification here. The first is to assume that the dropout process is sequentially ignorable (i.e. γ=0 ) and so the outcome $Y_{j}$ at visit j is not included in the logistic model for estimating the MLE weights. Note that these MLE weights are also used as the initial weights for calibration. The second is functional form model misspecification in the dropout model caused by including transformations $Z=\{Z_{1},Z_{2},Z_{3},Z_{4}\}$ of $V=\{V_{1},V_{2},V_{3},V_{4}\}$ in the model, rather than V itself (see Table 1).

We will investigate the impact of combinations of both types of misspecification in the simulations. When $Y_{j}$ is included in the dropout model, we use the true value for the sensitivity parameter γ when the correct covariates V and $Y_{\;j-1}$ are included. When the transformed covariates Z are instead included, we fix γ at its limiting value calculated by fitting a dropout model with the transformed covariates, $Y_{\;j-1}$ and $Y_{j}$ to a huge data set (sample size $n=1.28\times 10^{8}$ ).

When $Y_{j}$ is included, we estimate the MLE weights and calibrated weights using the methods described in Section 2. When $Y_{j}$ is omitted, we estimate the MLE weights by fitting a standard logistic regression model for the discrete-time hazard of dropout by maximum likelihood and then apply the calibration. In addition, following Cao et al.,^26^ we scale the MLE weights so that they sum to nT , the total number of follow-up observations in the absence of dropout. This scaling is expected to improve the stability of the IPCWE by prohibiting extremely large weights. Note that the baseline visits have weights of ones (i.e. no weighting is applied).

In total, we will evaluate the performance of the IPCWEs for $\left(\beta_{0},\beta_{1}\right)$ using three sets of weights, (a) the unscaled MLE weights, (b) the scaled MLE weights and (c) calibrated weights, under the following scenarios:

1. Selection function $\gamma Y_{j}$ omitted, correct covariates V and previous outcome $Y_{\;j-1}$ included;
2. Selection function $\gamma Y_{j}$ omitted, transformed covariates Z and previous outcome $Y_{\;j-1}$ included;
3. Selection function $\gamma Y_{j}$ included, correct covariates V and previous outcome $Y_{\;j-1}$ included;
4. Selection function $\gamma Y_{j}$ included, transformed covariates Z and previous outcome $Y_{\;j-1}$ included.

For covariates in calibrated restrictions, we include the visit number j (treated as a continuous variable), the baseline covariates ( V or Z ), the previous outcome $Y_{\;j-1}$ , and their interactions with the visit number j . For comparison, we also perform the analysis that uses the complete data and the naïve analysis that uses the observed data without weighting.

#### 3.2.2 Results

Table 2 summarizes the results of the first simulation study. When $\gamma Y_{j}$ is omitted and only baseline covariates (either in correct forms or in transformations) and $Y_{\;j-1}$ are included, the IPCWEs using the MLE weights and calibrated weights all have non-negligible biases. The IPCWEs using the MLE weights sometimes have similar or smaller biases than the IPCWEs using calibrated weights, but their root MSEs are much larger than those of the IPCWEs using calibrated weights. This is possibly because large positive and negative differences from the true parameter values can be cancelled out when averaging across samples to quantify the empirical bias but would manifest by the magnitude of MSEs. In terms of MSEs, the IPCWEs with calibrated weights are all smaller than their counterparts with the MLE weights. This is not surprising in view of the theoretical findings in Tan^11^ on the impact of calibration by covariate balancing on reducing the MSEs of inverse probability weighted estimators. The IPCWEs using scaled MLE weights have smaller MSEs than the IPCWEs using unscaled MLE weights, especially when transformed covariates are included.

**Table 2.** Bias, empirical standard deviation (SD) and root mean squared error (MSE) for IPCWEs of $\left(\beta_{0},\beta_{1}\right)$ in the first simulation study when dropout is sequentially non-ignorable. MLE weights: unscaled MLE weights; SMLE weights: scaled MLE weights; CMLE weights: calibrated weights. The naïve analysis without weighting and the analysis based on complete data are also presented.

		n=200			n=500			n=1000			n=2000		
		Bias	SD	MSE	Bias	SD	MSE	Bias	SD	MSE	Bias	SD	MSE
** $Y_{j}$ not included, $Y_{\;j-1}$ included**													
*Correct covariates*													
MLE	$\beta_{0}$	− 0.43	3.80	3.83	− 0.22	2.58	2.59	− 0.28	1.88	1.91	− 0.14	1.53	1.54
	$\beta_{1}$	0.76	0.97	1.24	0.60	0.86	1.05	0.59	0.62	0.85	0.52	0.65	0.83
SMLE	$\beta_{0}$	− 0.45	3.76	3.79	− 0.24	2.56	2.57	− 0.29	1.86	1.89	− 0.15	1.50	1.51
	$\beta_{1}$	0.77	0.96	1.23	0.60	0.85	1.04	0.59	0.61	0.85	0.52	0.65	0.83
CMLE	$\beta_{0}$	0.30	2.74	2.76	0.23	1.75	1.77	0.18	1.23	1.24	0.20	0.90	0.92
	$\beta_{1}$	0.36	0.31	0.47	0.34	0.22	0.41	0.33	0.16	0.37	0.33	0.13	0.35
*Transformed covariates*													
MLE	$\beta_{0}$	− 1.04	4.81	4.92	− 0.73	5.18	5.23	− 0.48	4.97	4.99	− 0.49	5.15	5.18
	$\beta_{1}$	0.47	1.55	1.62	0.11	1.80	1.80	-0.09	2.03	2.03	− 0.15	2.38	2.38
SMLE	$\beta_{0}$	− 1.06	4.63	4.75	− 0.75	4.52	4.58	− 0.56	4.26	4.29	− 0.53	4.34	4.37
	$\beta_{1}$	0.47	1.55	1.62	0.11	1.74	1.74	-0.08	1.95	1.95	− 0.14	2.30	2.31
CMLE	$\beta_{0}$	0.18	2.80	2.81	0.12	1.84	1.84	0.11	1.37	1.38	0.13	1.25	1.26
	$\beta_{1}$	0.37	0.49	0.61	0.23	0.37	0.44	0.11	0.40	0.41	0.04	0.60	0.60
** $Y_{\;j-1}$ included, $\gamma Y_{j}$ included with fixed γ **													
*Correct covariates*													
MLE	$\beta_{0}$	− 0.69	3.76	3.82	− 0.44	2.83	2.87	− 0.50	2.09	2.15	− 0.31	1.88	1.90
	$\beta_{1}$	0.54	1.04	1.17	0.31	1.04	1.09	0.28	0.76	0.81	0.16	0.84	0.86
SMLE	$\beta_{0}$	− 0.72	3.75	3.82	− 0.48	2.78	2.82	− 0.52	2.06	2.12	− 0.32	1.83	1.86
	$\beta_{1}$	0.54	1.04	1.17	0.32	1.03	1.07	0.28	0.75	0.80	0.16	0.84	0.85
CMLE	$\beta_{0}$	− 0.03	2.77	2.77	− 0.08	1.78	1.78	− 0.12	1.25	1.25	− 0.08	0.92	0.93
	$\beta_{1}$	0.16	0.34	0.38	0.11	0.24	0.27	0.08	0.19	0.21	0.06	0.16	0.17
*Transformed covariates*													
MLE	$\beta_{0}$	− 0.98	4.28	4.39	− 0.71	3.84	3.91	− 0.63	3.83	3.88	− 0.74	4.41	4.47
	$\beta_{1}$	0.35	1.46	1.50	0.03	1.63	1.63	− 0.14	1.81	1.81	− 0.16	2.12	2.12
SMLE	$\beta_{0}$	− 1.04	4.22	4.34	− 0.77	3.68	3.75	− 0.68	3.59	3.66	− 0.77	3.95	4.02
	$\beta_{1}$	0.36	1.45	1.49	0.03	1.60	1.60	− 0.13	1.76	1.77	− 0.15	2.05	2.06
CMLE	$\beta_{0}$	− 0.03	2.77	2.77	− 0.05	1.81	1.81	− 0.06	1.29	1.29	− 0.01	0.98	0.98
	$\beta_{1}$	0.16	0.48	0.50	− 0.01	0.37	0.37	− 0.15	0.35	0.38	− 0.25	0.32	0.41
**Naïve analysis**													
	$\beta_{0}$	1.31	2.77	3.07	1.26	1.75	2.16	1.19	1.25	1.73	1.24	0.89	1.53
	$\beta_{1}$	2.14	0.51	2.20	2.15	0.32	2.17	2.16	0.23	2.17	2.15	0.16	2.16
**Complete data**													
	$\beta_{0}$	0.05	2.66	2.66	− 0.00	1.68	1.68	− 0.07	1.19	1.19	− 0.03	0.86	0.87
	$\beta_{1}$	− 0.00	0.09	0.09	− 0.00	0.06	0.06	0.00	0.04	0.04	0.00	0.03	0.03

When the selection function $\gamma Y_{j}$ is included, the IPCWEs with correct covariates and calibrated weights have both smaller biases and smaller MSEs than their counterparts with the MLE weights. It is noteworthy that the IPCWEs with the MLE weights still have a moderate amount of finite sample bias even when the dropout model has been correctly specified, although their biases and MSEs decrease as the sample size increases. This is perhaps due to the relatively extreme specification of the dropout model (adapted from the simulation set-up in Kang and Schafer^3^), which causes the MLE weights to have large variability. The IPCWEs with calibrated weights also have a small amount of bias, but for $\beta_{0}$ the magnitude of bias is comparable to the analysis using the complete data, and for $\beta_{1}$ the bias is decreasing as the sample size increases. On the other hand, with transformed covariates (and hence model misspecification), the MSEs of the IPCWEs of $\beta_{1}$ using the MLE weights increase as the sample size increases. This happens possibly because the probability that a simulated dataset contains an extreme MLE weight increases as the sample size increases; see Robins et al.^27^ (pp. 553–4) for more details. In contrast, the IPCWEs with calibrated weights do not exhibit this undesirable property and show more robustness to the functional form misspecification in the set-up of the first simulation study.

#### 3.2.3 Summary

In all scenarios of the first simulation study, the IPCWEs with calibrated weights perform uniformly better in terms of MSEs than the IPCWEs with the MLE weights. When the rate of missingness is high ( 43% ), the dropout model is correctly specified (i.e. with correct selection function and correct covariates) and sample sizes are larger ( n=1000,2000 ), the IPCWEs with calibrated weights still have MSEs that are not very far from the MSEs of the estimators based on the complete data, demonstrating the remarkable gain in efficiency that can be achieved by calibrating IPCWEs.

### 3.3 Coverage of 95% confidence intervals based on bootstrap, jackknife or sandwich variance estimator when the dropout process is sequentially ignorable

#### 3.3.1 Setup

Since the first simulation study has demonstrated the better performance of the IPCWEs with calibrated weights under non-ignorable dropout when the selection function and correct covariates are used, for simplicity, in the second simulation study, we focus on the setting where the dropout process is sequentially ignorable (i.e. γ=0 ) and correct baseline covariates V and $Y_{\;j-1}$ are included.

We will compare the empirical coverage probabilities of the following CIs:

1. Bootstrap CIs with calibrated weights, fixing the initial weights at those estimated from the original data.
2. Bootstrap CIs with calibrated weights, re-estimating the initial weights for each bootstrap sample.
3. Bootstrap CIs with unscaled MLE weights.
4. Bootstrap CIs with scaled (to sum to nT ) MLE weights.
5. For n=200,500 , jackknife CIs with calibrated weights, fixing the initial weights at those estimated from the original data.
6. For n=200,500 , jackknife CIs with calibrated weights, re-estimating the initial weights for each jackknife sample.
7. For n=200,500 , jackknife CIs with unscaled MLE weights.
8. For n=200,500 , jackknife CIs with scaled MLE weights.
9. CIs based on sandwich variance estimator and unscaled MLE weights.
10. CIs based on sandwich variance estimator and scaled MLE weights.
11. CIs based on sandwich variance estimator and calibrated weights.

The MLE weights were calculated by fitting a standard logistic regression model for the dropout hazard given the correct baseline covariates V and $Y_{\;j-1}$ . For bootstrap CIs (1)–(4), 500 bootstrap samples with replacement were generated, where patients were resampling units, and the percentile method was used. For the smaller sample sizes n=200,500 , we constructed jackknife CIs (5)–(8) by using the jackknife standard error. For CIs (9)–(11), based on the sandwich estimator of the variance, we used the R package geepack to apply GEEs with the independence working correlation structure. The empirical coverage probabilities of the 95% CIs were calculated as the proportions of simulations in which these CIs include the true values of $\beta_{0},\beta_{1}$ .

#### 3.3.2 Results

Table 3 presents the coverage probabilities of the 95% CIs based on non-parametric bootstrap, jackknife and sandwich estimator. The bootstrap CIs with calibrated weights all achieved better coverage than their counterparts with the MLE weights, and their coverage is close to the 95% nominal level. This is regardless of whether or not the initial weights were re-estimated for each bootstrap sample. This finding confirms that fixing the initial weights has minimal impact on the coverage of the bootstrap CIs with calibrated weights. Overall, the bootstrap CIs with the scaled MLE weights have slightly better coverage than their counterparts with unscaled weights, but the improvement is minimal.

**Table 3.** Coverage probabilities (%) of 95% confidence intervals (CIs) based on non-parametric bootstrap, jackknife (only for n=200,500 ) and sandwich variance estimator for $\left(\beta_{0},\beta_{1}\right)$ in the second simulation study when dropout is sequentially ignorable. MLE weights: unscaled MLE weights; SMLE weights: scaled MLE weights; CMLE weights: calibrated weights.

		n=200	n=500	n=1000	n=2000
Bootstrap, CMLE weights, fixing initial weights	$\beta_{0}$	94.3	94.0	94.7	94.7
	$\beta_{1}$	94.7	94.6	95.0	95.0
Bootstrap, CMLE weights, re-estimating initial weights	$\beta_{0}$	94.3	93.8	95.0	94.5
	$\beta_{1}$	94.7	94.5	95.5	93.9
Bootstrap, MLE weights	$\beta_{0}$	86.5	74.5	61.6	44.0
	$\beta_{1}$	91.2	91.4	92.3	90.8
Bootstrap, SMLE weights	$\beta_{0}$	86.8	75.9	64.3	46.5
	$\beta_{1}$	92.0	92.5	93.1	92.2
Jackknife, CMLE weights, fixing initial weights	$\beta_{0}$	94.7	94.7		
	$\beta_{1}$	95.0	95.4		
Jackknife, CMLE weights, re-estimating initial weights	$\beta_{0}$	94.3	94.2		
	$\beta_{1}$	93.2	93.8		
Jackknife, MLE weights	$\beta_{0}$	96.3	96.2		
	$\beta_{1}$	95.5	94.2		
Jackknife, SMLE weights	$\beta_{0}$	95.7	95.5		
	$\beta_{1}$	95.0	93.7		
Sandwich variance estimator, MLE weights	$\beta_{0}$	94.6	93.7	94.0	94.8
	$\beta_{1}$	92.3	90.5	89.9	88.4
Sandwich variance estimator, SMLE weights	$\beta_{0}$	94.7	93.8	94.0	94.8
	$\beta_{1}$	92.4	90.5	89.8	88.5
Sandwich variance estimator, CMLE weights	$\beta_{0}$	97.5	98.2	98.1	98.5
	$\beta_{1}$	100.0	100.0	99.9	100.0

The poor coverage of the bootstrap CIs with the MLE weights might be explained by the moderate amount of finite sample bias of the IPCWEs with the MLE weights. For example, as shown in the top part of Table 4, the IPCWEs with the MLE weights showed a moderate amount of empirical bias, especially for $\beta_{0}$ , while the IPCWEs with calibrated weights had negligible biases of similar magnitude to those from the analysis based on the complete data. On the other hand, the maximum likelihood estimates of regression coefficient parameters in the dropout model appear to be unbiased, as shown in the bottom part of Table 4. This suggests that the good performance of parameter estimators in the dropout model does not necessarily translate to the good performance of IPCWEs for parameters in the outcome model. Interestingly, the coverage of the bootstrap CIs of $\beta_{0}$ with the MLE weights is even worse when the sample size increases. This might be due to one or more extreme MLE weights being more likely to occur in bootstrap samples when the sample size is larger (e.g. n=2000 ).

**Table 4.** **Top:** Bias, empirical standard deviation (SD) and root mean squared error (MSE) for IPCWEs of $\beta_{0}$ and $\beta_{1}$ in the second simulation study. MLE weights: unscaled MLE weights; SMLE weights: scaled MLE weights; CMLE weights: calibrated weights. The naïve analysis without weighting and the analysis based on complete data (‘COMP’) are also presented. **Bottom:** Bias, empirical standard deviation (SD) and root mean squared error (MSE) of the intercept (‘Int.’) and regression coefficients of baseline covariates and the previous outcome in fitted logistic models for dropout.

		n=200			n=500			n=1000			n=2000		
		Bias	SD	MSE	Bias	SD	MSE	Bias	SD	MSE	Bias	SD	MSE
MLE	$\beta_{0}$	0.32	3.44	3.45	0.25	2.38	2.40	0.13	2.00	2.00	0.11	1.47	1.47
	$\beta_{1}$	− 0.22	0.91	0.93	− 0.14	0.77	0.78	− 0.09	0.71	0.72	− 0.07	0.54	0.54
SMLE	$\beta_{0}$	0.34	3.41	3.43	0.26	2.35	2.37	0.14	1.94	1.94	0.12	1.44	1.44
	$\beta_{1}$	− 0.22	0.90	0.93	− 0.14	0.76	0.77	− 0.09	0.70	0.71	− 0.07	0.53	0.53
CMLE	$\beta_{0}$	− 0.03	2.76	2.76	0.02	1.77	1.77	− 0.03	1.24	1.24	0.01	0.89	0.89
	$\beta_{1}$	0.02	0.27	0.28	0.01	0.19	0.19	0.01	0.14	0.14	0.00	0.11	0.11
Naïve	$\beta_{0}$	− 0.83	2.82	2.94	− 0.83	1.78	1.96	− 0.87	1.27	1.53	− 0.83	0.89	1.22
	$\beta_{1}$	− 1.44	0.46	1.51	− 1.45	0.30	1.48	− 1.45	0.21	1.46	− 1.45	0.15	1.46
COMP	$\beta_{0}$	− 0.02	2.70	2.70	0.01	1.72	1.72	− 0.03	1.21	1.21	0.01	0.87	0.87
	$\beta_{1}$	0.00	0.09	0.09	− 0.00	0.06	0.06	0.00	0.04	0.04	− 0.00	0.03	0.03
													
	Int.	0.00	0.12	0.12	0.00	0.07	0.07	0.00	0.05	0.05	0.00	0.04	0.04
	$V_{1}$	− 0.03	0.23	0.23	− 0.01	0.15	0.15	− 0.01	0.10	0.10	− 0.00	0.07	0.07
	$V_{2}$	0.01	0.14	0.14	0.00	0.09	0.09	0.00	0.06	0.06	0.00	0.04	0.04
	$V_{3}$	− 0.01	0.14	0.14	− 0.00	0.09	0.09	− 0.00	0.06	0.06	− 0.00	0.04	0.04
	$V_{4}$	− 0.01	0.14	0.14	− 0.00	0.09	0.09	− 0.00	0.06	0.06	− 0.00	0.04	0.04
	$Y_{\;j-1}$	0.02	0.24	0.24	0.01	0.15	0.15	0.00	0.11	0.11	0.00	0.08	0.08

The coverage of the jackknife CIs is similar with different sets of weights. This might be because extreme MLE weights are less likely to occur in jackknife samples, as only one patient’s data are removed in each jackknife sample.

For $\beta_{0}$ , CIs based on the sandwich estimator and the MLE weights have better coverage than the bootstrap CIs using the MLE weights. But for $\beta_{1}$ , CIs based on the sandwich estimator and the MLE weights have slightly poorer coverage than the bootstrap CIs using the MLE weights. In contrast, CIs based on the sandwich estimator and calibrated weights are very conservative, with 97.5−100% coverage. This is because the uncertainty of calibrated weights is ignored.

#### 3.3.3 Summary

The bootstrap CIs using the MLE weights performed poorly in this simulation study, while the jackknife CIs and CIs based on sandwich variance estimator using the MLE weights performed better, possibly because they were less likely to be impacted by extreme MLE weights generated during re-sampling. Bootstrap and jackknife CIs using calibrated weights performed well, but CIs based on the sandwich estimator using calibrated weights were conservative. There was essentially no difference in terms of coverage when the initial weights were re-estimated or not re-estimated for each bootstrap/jackknife sample. Therefore, for computational efficiency, it is reasonable to fix the initial weights when constructing bootstrap/jackknife CIs with calibrated weights, especially if the model for estimating the initial weights is complex and slow to fit.

## 4 Analysis of the SLICC cohort data

### 4.1 The longitudinal outcome, covariates and secondary sources of missing data

The outcome of interest in our analysis is the physical component summary (PCS) score from the SF-36 questionnaire completed by the SLICC patients at their study assessment visits. The PCS includes four subscales of physical functioning (10 items), role-physical (4 items), bodily pain (2 items), and general health (5 items), and is standardized to range between 0 and 100. Following Hanly et al.,^19^ we treat the PCS as a continuous variable. The main covariate of interest is patient’s time-varying NP status at annual study assessments. This is a categorical variable with three levels, which are, in order of increasing severity: (i) patient has not yet had an NP event; (ii) patient has had an NP event, but not yet a CerVE attributable to SLE; and (iii) patient has had a CerVE attributable to SLE. See Hanly et al. ^19^ for the definition of CerVEs attributable to SLE. We used (iii) as the reference category. Following Hanly et al.,^19^ other covariates for the outcome model include: assessment visit number (including linear and quadratic terms); sex; age at SLE diagnosis; groups defined by race/ethnicity/location; post-secondary education; the SLE Disease Activity Index 2000 (SLEDAI-2K) at the current assessment; SLICC/ACR Damage Index (SDI) excluding the NP component at the current assessment; corticosteroids, antimalarials and immuno-suppressant use since last assessment visit.

Besides the loss to follow-up, there were secondary sources of missing data which resulted in intermittent missing data in the PCS and missing data in other covariates such as education, SLEDAI-2K and SDI. It was not uncommon for patients to miss more than one annual assessment visit and then came back for later assessments, which resulted in a moderate amount of intermittent missing data in the PCS (19.4% out of 12,889 assessment visits). When there was one intermittent missing PCS value, the last observed value was carried forward; otherwise the patient was treated as having dropped out immediately before the first of the two or more consecutive missed visits, and any subsequent visits were ignored. Missing PCS and SLEDAI-2K values at enrolment (visit 0) were imputed as the patient’s values at the first follow-up visit (visit 1), if these values were available; patients with missing PCS and SLEDAI-2K values at visits 0 and 1 were excluded from the analysis. Other intermittent missing SLEDAI-2K values were imputed by ‘last observation carry forward’. In addition, we categorized SDI values as 0, 1, 2, 3 and ≥4 and created a separate category for missing SDI. This is because SDI was only available for recording after certain conditions were present for at least 6 months and therefore there were a lot of missing SDI values at enrolment in patients who had only recently been diagnosed with SLE. For all other covariates, we used ‘last observation carry forward’ to impute the missing values. Finally, we excluded 87 patients with missing education information. This left 1574 patients in the analysis. We administratively censor the follow-up at the earliest of visit 10 and 10 December 2015. As a result, the maximum number of visits that the 1574 SLICC patients could potentially make before 10 December 2015 was 12,887, which is less than 1574×11=17,314 . Of these 12,887 potential visits, 8901 had PCS values that were either observed or imputed as described above.

### 4.2 The dropout model and estimated weights

We first fit the dropout model of (3) using MLE and assuming that the dropout process is sequentially ignorable, that is, γ=0 . The set of baseline covariates listed in Section 4.1 is included in this model. For the PCS and time-varying covariates (including NP status, SLEDAI, SDI scores and medication use), the most recent value is used. All continuous variables are standardized. Linear and quadratic effects of continuous variables are included in the model initially, but quadratic effects are removed if they are not significantly associated with the dropout hazard. Table 5 presents the estimates, standard errors, 95% CIs and p-values of Wald tests for regression coefficients in the fitted dropout model. Note that positive regression coefficient estimates indicate smaller probabilities of dropping out. Dropout is significantly associated with race/location, antimalarial use, SDI scores, and age at SLE diagnosis (borderline significant). Since SLE patients taking antimalarials usually have milder disease activities, this might explain why these patients were more likely to remain in the study. By definition, patients with higher SDI scores have had higher disease activities, which have led to permanent damage to various organs. Thus, it is not surprising that these patients were less likely to remain in the study. There was a lot of variation across race/location groups in terms of dropout hazards, which could partly relate to differences in disease severity across race/ethnicity groups and differences across locations in disease management practices. However, after adjusting for the above covariates, the dropout hazard does not appear to be associated with the previous PCS scores.

**Table 5.** Fitted model for the dropout process in the SLICC data. LL: lower limit of 95% CI; UL: upper limit of 95% CI.

	Estimate	Std. Error	LL	UL	*p*-value
Intercept	2.19	0.24	1.71	2.66	<0.01
Visit					<0.01
(linear term)	1.23	0.49	0.27	2.18	
(quadratic term)	− 0.45	0.54	− 1.51	0.61	
Male	0.03	0.12	− 0.20	0.26	0.82
Age at SLE diagnosis					0.06
(linear term)	0.09	0.04	0.00	0.18	
(quadratic term)	− 0.06	0.03	− 0.12	− 0.00	
Post-secondary education (yes)	0.05	0.08	− 0.10	0.20	0.51
Race/location groups (vs. EU/Canada Caucasian)					<0.01
US Caucasian	− 1.56	0.11	− 1.78	− 1.35	
Hispanic	− 1.06	0.12	− 1.29	− 0.83	
US African	− 1.62	0.13	− 1.87	− 1.37	
Other African	0.07	0.17	− 0.26	0.39	
Asian	− 0.42	0.12	− 0.65	− 0.18	
Other races	− 0.69	0.19	− 1.05	− 0.33	
Corticosteroids use (yes)	0.05	0.08	− 0.12	0.21	0.57
Antimalarial use (yes)	0.17	0.08	0.02	0.33	0.03
Immuno-suppressant use (yes)	0.14	0.08	− 0.02	0.30	0.09
SLEDAI	0.05	0.04	− 0.02	0.12	0.19
SDI w/o NP					0.01
1 vs. 0	− 0.22	0.11	− 0.43	− 0.00	
2 vs. 0	0.05	0.15	− 0.24	0.35	
3 vs. 0	− 0.38	0.20	− 0.77	0.00	
>=4 vs. 0	− 0.69	0.22	− 1.12	− 0.25	
NA vs. 0	− 0.11	0.12	− 0.35	0.12	
NP status					0.47
Other NP w/o CerVE vs. CerVE	0.15	0.20	− 0.24	0.55	
No NP vs. CerVE	0.07	0.20	− 0.32	0.46	
PCS at the previous visit	0.03	0.04	− 0.04	0.11	0.39

We used the fitted dropout model to obtain the initial weights and then applied the calibration procedure in Section 2.3. To accommodate the administrative censoring due to staggered entry to the SLICC cohort, we slightly modify the calibration restrictions in (6) and (7) by replacing T with $T_{i}$ , the maximum number of potential follow-up visits a patient could make before the study end. To make a fair comparison, we scale the MLE weights to make $\sum_{i=1}^{n}\sum_{\;j=1}^{T_{i}}R_{ij}W_{ij}^{C}(\hat{\alpha },\gamma )=\sum_{i=1}^{n}T_{i}$ , while the baseline visits have weights of ones. Note that here we assume that the missingness due to administrative censoring is completely at random, so that $E(Y_{ij}|X_{ij})=E(Y_{ij}|X_{ij},T_{i}\geq j)$ . We first estimated the calibrated weights by including all variables listed in Table 5 in the calibration restrictions. In addition, we obtained calibration weights by further adding the interactions between the visit number and baseline and time-varying covariates, as recommended in Section 2.3 and implemented in the simulation studies. The results for the outcome model and the sensitivity analysis were very similar for the two sets of calibrated weights. Therefore here we only present the results from applying the first set of calibrated weights.

**Figure 1.**
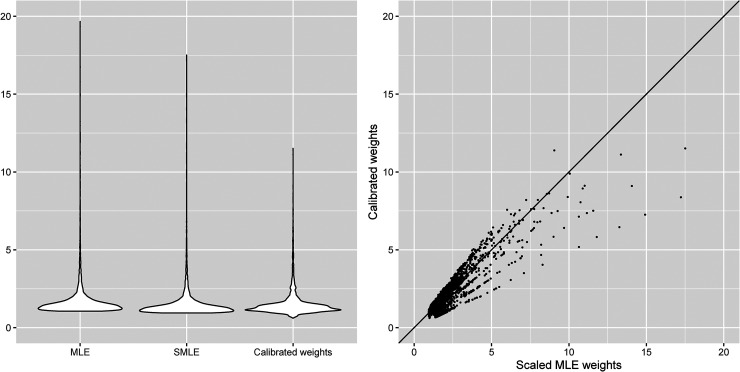
Violin plots and scatterplot of the MLE weights (MLE: unscaled MLE weights; SMLE: scaled MLE weights) and calibrated weights assuming sequential ignorability of the dropout process in the SLICC data.

Figure 1 presents the violin plots of the unscaled MLE weights, scaled MLE weights and calibrated weights for the follow-up visits, as well as the scatterplot of the scaled MLE weights and calibrated weights. The minimum/maximum values of the unscaled MLE weights, scaled MLE weights and calibrated weights are 1.058/19.663, 0.942/17.508 and 0.616/11.514, respectively. It is clear from Figure 1 that the calibrated weights are less variable than the MLE weights. Overall, these weights appear to be not very extreme, which suggests that the positivity assumption is likely to hold.

Table 6 presents the summaries of the demographic variables in the target population and the weighted samples using the unscaled MLE weights, scaled MLE weights and calibrated weights. It is apparent that calibrated weights exactly balance the distributions of all the demographic variables in the target population and the weighted samples, but the MLE weights only seem to balance age at SLE diagnosis well.

**Table 6.** Demographics summaries in the target population (Target) and the weighted samples using the unscaled MLE weights (MLE), scaled MLE weights (SMLE) and calibrated weights (CMLE) in the SLICC data analysis.

	Target	MLE	SMLE	CMLE
Gender (number of observations before study cut-off)				
Female	11524	12794.99	11546.33	11524
Male	1363	1484.25	1340.67	1363
Race/location (number of observations before study cut-off)				
EU/Canada Caucasian	4609	5153.05	4651.06	4609
US Caucasian	1778	1907.95	1722.55	1778
Hispanic	1785	2031.99	1832.45	1785
US African	1121	1183.01	1068.61	1121
Other African	987	1104.38	996.62	987
Asian	2105	2354.79	2124.59	2105
Other	502	544.06	491.13	502
Age at SLE diagnosis				
Mean	34.67	34.57	34.57	34.67
Standard deviation	13.30	13.27	13.27	13.30

### 4.3 Results for the outcome model

Columns 1–9 in Table 7 present the point estimates and bootstrap SEs of regression coefficients for the naïve analysis without weighting, and IPCWEs using the MLE weights and calibrated weights assuming sequential ignorability. For estimators with calibrated weights we note that the bootstrap SEs with and without recalculating the initial weights are almost identical.

**Table 7.** Point estimates (EST) and bootstrap standard errors (SE) of regression coefficients from the naïve analysis without weighting and IPCWEs with the MLE weights and calibrated weights. MLE (unscaled and scaled): weights based on maximum likelihood; CMLE: calibrated weights. SE1: bootstrap standard errors by recalculating the initial weights for calibrating weights; SE2: bootstrap standard errors by fixing the initial weights for calibrating weights. γ is the coefficient of the current longitudinal outcome in the dropout model, that is, the sensitivity parameter. For sensitivity analysis, we allow γ to vary at 1–4 times of the estimated coefficient (0.03) of the previous outcome $Y_{\;j-1}$ in the dropout model assuming sequential ignorability. Here we only present the result when γ=0.03 .

	Naïve		γ=0							γ=0.03						
			MLE (unscaled)		MLE (scaled)		CMLE			MLE (unscaled)		MLE (scaled)		CMLE		
	EST	SE	EST	SE	EST	SE	EST	SE1	SE2	EST	SE	EST	SE	EST	SE1	SE2
Intercept	38.06	1.57	38.57	1.74	38.51	1.73	38.23	1.71	1.72	38.63	1.75	38.57	1.74	38.29	1.72	1.72
Visit																
(linear term)	11.92	1.45	10.72	1.66	10.90	1.65	11.04	1.63	1.66	10.51	1.66	10.69	1.64	10.84	1.63	1.67
(quadratic term)	− 10.14	1.48	− 8.95	1.77	− 9.10	1.76	− 9.32	1.78	1.78	− 8.82	1.77	− 8.97	1.76	− 9.20	1.78	1.78
NP status																
Other NP w/o CerVE vs. CerVE	1.59	1.46	1.72	1.57	1.72	1.56	1.96	1.57	1.56	1.70	1.58	1.70	1.57	1.92	1.57	1.57
No NP vs. CerVE	5.03	1.42	4.91	1.53	4.90	1.52	5.13	1.53	1.53	4.89	1.54	4.88	1.53	5.10	1.54	1.54
Male	3.21	0.79	3.41	0.86	3.40	0.86	3.28	0.86	0.87	3.41	0.87	3.40	0.86	3.28	0.86	0.87
Age at SLE diagnosis	− 2.63	0.25	− 2.52	0.31	− 2.51	0.30	− 2.49	0.30	0.30	− 2.53	0.31	− 2.53	0.30	− 2.50	0.30	0.30
Race/location groups																
(vs. EU/Canada Caucasian)																
US Caucasian	− 3.38	0.96	− 3.35	1.19	− 3.35	1.18	− 3.48	1.13	1.15	− 3.44	1.18	− 3.45	1.17	− 3.58	1.12	1.15
Hispanic	3.13	0.78	3.93	0.88	3.91	0.87	3.74	0.85	0.85	3.91	0.88	3.89	0.88	3.73	0.85	0.86
US African	− 0.22	1.04	0.65	1.23	0.61	1.22	0.48	1.17	1.17	0.58	1.22	0.54	1.21	0.42	1.17	1.17
Other African	− 0.92	0.95	− 0.50	0.98	− 0.52	0.97	− 0.61	0.96	0.96	− 0.49	0.98	− 0.51	0.97	− 0.60	0.96	0.96
Asian	3.42	0.69	3.64	0.74	3.62	0.74	3.52	0.73	0.73	3.63	0.74	3.62	0.74	3.52	0.73	0.73
Other races	− 1.68	1.30	− 1.33	1.42	− 1.33	1.41	− 1.30	1.38	1.38	− 1.39	1.43	− 1.39	1.42	− 1.34	1.39	1.39
Post-secondary education (yes)	1.38	0.52	1.06	0.55	1.07	0.54	1.16	0.53	0.54	1.04	0.55	1.05	0.55	1.14	0.54	0.54
SLEDAI	− 0.80	0.16	− 0.74	0.18	− 0.75	0.18	− 0.72	0.18	0.18	− 0.73	0.18	− 0.74	0.18	− 0.72	0.18	0.18
SDI w/o NP																
1 vs. 0	− 1.66	0.60	− 1.86	0.72	− 1.87	0.72	− 1.87	0.71	0.70	− 1.86	0.72	− 1.87	0.71	− 1.87	0.71	0.70
2 vs. 0	− 1.49	0.88	− 1.13	1.05	− 1.15	1.05	− 1.18	1.02	1.01	− 1.18	1.05	− 1.19	1.05	− 1.21	1.02	1.01
3 vs. 0	− 3.30	1.27	− 3.02	1.25	− 3.02	1.25	− 3.45	1.27	1.25	− 3.01	1.25	− 3.01	1.25	− 3.44	1.27	1.25
>=4 vs. 0	− 5.13	1.89	− 3.54	1.82	− 3.55	1.82	− 3.57	1.84	1.86	− 3.64	1.82	− 3.64	1.82	− 3.65	1.85	1.87
NA vs. 0	− 1.36	0.44	− 1.27	0.47	− 1.25	0.46	− 1.28	0.45	0.45	− 1.28	0.47	− 1.26	0.46	− 1.28	0.45	0.45
Corticosteroids use (yes)	− 3.56	0.48	− 4.35	0.56	− 4.32	0.55	− 4.26	0.53	0.53	− 4.35	0.56	− 4.31	0.55	− 4.26	0.53	0.53
Antimalarial use (yes)	0.04	0.48	− 0.00	0.54	0.00	0.53	0.10	0.53	0.53	− 0.01	0.54	0.00	0.53	0.11	0.53	0.53
Immuno-suppressant use (yes)	− 0.84	0.47	− 0.75	0.55	− 0.74	0.55	− 0.73	0.52	0.52	− 0.76	0.55	− 0.75	0.55	− 0.74	0.52	0.52

We focus on the main effect of interest, the ‘NP status’ effect. The effect of ‘other NP events without CerVEs’ vs. ‘CerVEs’ was increased when IPCW was applied, compared with the naïve analysis. This increase was most prominent when IPCW with calibrated weights was used. The effect of ‘No NP’ vs. ‘CerVEs’ was similar across different estimators. Nevertheless, the overall conclusions about the effects of ‘NP status’ remain the same: patients who had any previous NP events had lower PCS scores on average than patients who did not have any previous NP events. There is no evidence that the PCS scores differed between patients with any SLE-attributable CerVEs and patients with other NP events but without SLE-attributable CerVEs. In addition, there were fairly large changes in estimates of the effects of visit, race/location groups and corticosteroids use. For example, the negative effect of corticosteroids use on PCS was increased by one SE more when IPCW with calibrated weights was applied compared with the naïve analysis, which suggests stronger evidence for the negative association between corticosteroids use and patients’ HRQoL.

### 4.4 Sensitivity analysis

**Figure 2.**
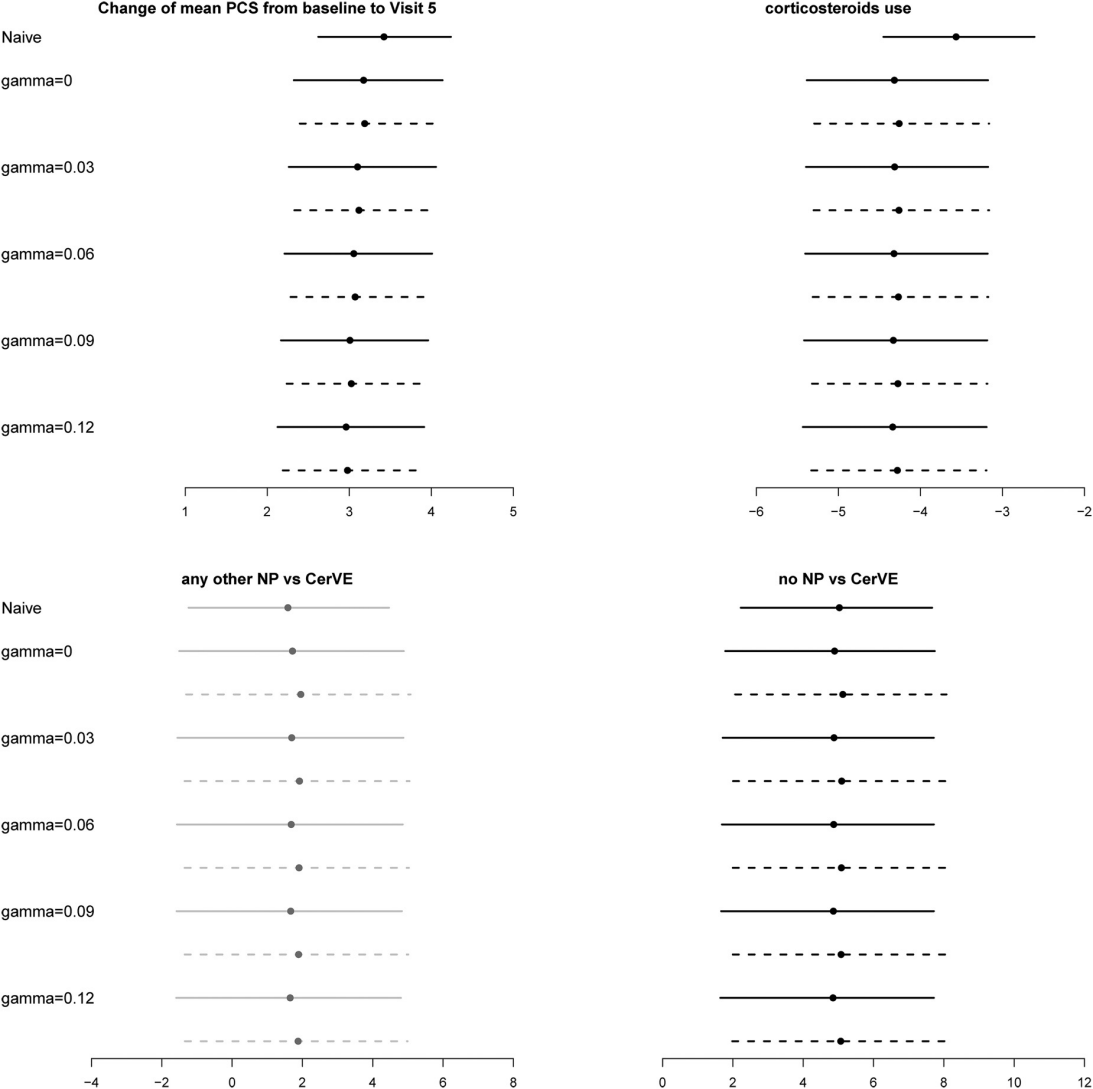
Estimated regression coefficients and 95% bootstrap confidence intervals for the SLICC data. Top left panel: changes of mean physical summary score (PCS) from baseline to visit 5. Top right panel: effect of corticosteroids use on PCS. Bottom panels: the long-term effect of the occurrence of cerebrovascular (CerVE) events or any other neuropsychiatric (NP) events on PCS. Dotted line: results from calibrated weights; solid lines: results from scaled MLE weights. The estimated effects with 95% CI covering zero and not covering zero are in grey and black, respectively. γ is the coefficient of the current longitudinal outcome in the dropout model, that is, the sensitivity parameter. For sensitivity analysis, we allow γ to vary from one to four times of the coefficient estimate of the previous outcome $Y_{\;j-1}$ in the dropout model assuming sequential ignorability.

For sensitivity analysis, we let γ vary from one to four times the estimated coefficient of the previous PCS (0.03) in the dropout model assuming sequential ignorability. That is, γ=0.03,0.06,0.09,0.12 . This choice to restrict to positive γ is based on the belief that patients with higher PCS scores were more likely to remain in the study. Columns 10–16 of Table 7 present the point estimates and SEs of IPCWEs when γ=0.03 . It would be clearer to use graphics for assessing the sensitivity of the point estimates and 95% bootstrap CIs to the value of γ . In Figure 2, we plot the estimates and CIs for the effects of visit (represented by the change in mean PCS from baseline to visit 5), corticosteroids use and NP status obtained from the naïve analysis and from the IPCWEs assuming different values of γ . For estimators with calibrated weights, the bootstrap CIs by re-estimating and fixing the initial weights are almost identical. Here we only present the result with initial weights re-estimated.

The reduced visit effect in the analyses with IPCW is obvious compared with the naïve analysis. This is expected because patients with higher PCS are more likely to remain in the study. As discussed above, there are slight differences between estimated NP status effects from the IPCWEs using MLE weights and those using the calibrated weights. However, the main conclusions about the effect of NP status on PCS are similar across different analyses with IPCW and consistent with the findings in Hanly et al.^19^

## 5 Conclusion and discussion

In this paper, we have proposed a sensitivity analysis approach for IPCWEs with calibrated weights under non-ignorable dropout. Simulation studies showed that IPCWEs using calibrated weights performed uniformly better than IPCWEs using weights estimated by maximum likelihood, including in settings with model misspecification. It was also shown that bootstrap and jackknife CIs based on calibrated weights performed well, but CIs based on the sandwich variance estimator and calibrated weights were conservative. Using the simple technique of fixing the initial set of weights before calibration to those from the original data made no difference to the coverage of bootstrap/jackknife CIs. This is particularly useful to speed up the computation when conducting sensitivity analyses since the analysis needs to be repeated for each set of values considered for the sensitivity parameters. The computational efficiency of the proposed sensitivity analysis approach, together with the better performance of the calibrated IPCWEs, will hopefully promote more widespread use of IPCW in practice.

The proposed methods can be extended to handle intermittent missingness. Vansteelandt et al.^17^ and Wen and Seaman^18^ have described sensitivity analysis approaches for non-monotone missingness using inverse probability weights without calibration. Their models can be used to estimate the initial weights before calibration. Then the calibration restriction in equation (6) can be modified to

(10) $$\sum_{i=1}^{n}\sum_{\;j=1}^{T}\left\{R_{ij}W_{ij}^{C}(\lambda )-1\right\}\phi_{j}({\bar{O}}_{i,j-1})=0$$

where $R_{ij}$ is the indicator of whether the outcome is observed at visit j for patient i , ${\bar{O}}_{i,j-1}=(V_{i},X_{i0},Y_{i0},R_{i1},R_{i1}X_{i1},R_{i1}Y_{i1},\ldots ,R_{i,j-1},R_{i,j-1}X_{i,j-1},R_{i,j-1}Y_{i,j-1})$ and $\phi_{j}({\bar{O}}_{i,j-1})$ is a function of ${\bar{O}}_{i,j-1}$ that has the same dimension of λ . This modified calibration restriction is very similar to the existing balancing conditions proposed for handling missingness in the cross-sectional settings,^9^ except that it is now aggregated over time.

In our simulation studies, we have demonstrated the better performance of the IPCWEs with calibrated weights than the IPCWEs with the MLE weights when the dropout model is misspecified. However, it would be interesting to investigate whether steps to reduce the risk of model misspecification when estimating the initial weights might improve the calibrated IPCWEs. Data adaptive methods are useful for this purpose. In addition, they might help to stabilize the calibrated weights, in light of the recently established connection between minimum dispersion approximate balance weights and penalized estimation of propensity scores using LASSO.^28^ In future research, we will explore the method of sieves^29^ for estimating the initial weights because it allows flexible data-adaptive estimation but retains the usual root- n consistency under regularity conditions and the validity of bootstrap/jackknife CIs.

## Supplemental Material

sj-zip-2-smm-10.1177_09622802221090763 - Supplemental material for Sensitivity analysis for calibrated inverse probability-of-censoring weighted estimators under non-ignorable dropout Supplemental material, sj-zip-2-smm-10.1177_09622802221090763 for Sensitivity analysis for calibrated inverse probability-of-censoring weighted estimators under non-ignorable dropout by Li Su, Shaun R Seaman and Sean Yiu in Statistical Methods in Medical Research

sj-zip-3-smm-10.1177_09622802221090763 - Supplemental material for Sensitivity analysis for calibrated inverse probability-of-censoring weighted estimators under non-ignorable dropout Supplemental material, sj-zip-3-smm-10.1177_09622802221090763 for Sensitivity analysis for calibrated inverse probability-of-censoring weighted estimators under non-ignorable dropout by Li Su, Shaun R Seaman and Sean Yiu in Statistical Methods in Medical Research
