# Supplementary material for: Sensitivity analysis for calibrated inverse probability-of-censoring weighted estimators under non-ignorable dropout
Source: Stat Methods Med Res. 2022 Apr 12;31(7):1374–91. doi: 10.1177/09622802221090763 (PMC9253927; doi:10.1177/09622802221090763)
Supplement: sj-zip-2-smm-10.1177_09622802221090763 - Supplemental material for Sensitivity analysis for calibrated inverse probability-of-censoring weighted estimators under non-ignorable dropout [file sj-zip-2-smm-10.1177_09622802221090763.zip › Tutorial.pdf]

# A quick tutorial of implementing calibrated inverse probability of censoring weighted estimators in Su, Seaman and Yiu (2022)

Li Su

This is a quick tutorial to demonstrate how to implement the calibrated inverse probability of censoring weighted estimators (IPCWEs) and conduct sensitivity analyses for non-ignorable dropout proposed in Su, Seaman and Yiu (2022). Functions to be called in this tutorial are saved in *Functions.R*.

```
source('Functions.R') ## functions to be called
```

### 1. Data

A dataset was simulated based on the data generating mechanism described in Table 1 of Su, Seaman and Yiu (2022). The dropout process depends on four baseline covariates $\{X_1, X_2, X_3, X_4\}$, the previous outcome $Y_{j-1}$ and the current outcome $Y_j$. Specifically, let $\pi_j$ be 1 minus the discrete hazard of drop-out. It follows a logistic model

$$\text{logit}(\pi_j) = 2 - X_1 + 0.5X_2 - 0.25X_3 - 0.1X_4 + 0.2Y^*_{j-1} + Y^*_j,$$

where $Y^* = (Y - 210)/35$.

We are interested in estimating $\beta_0$ and $\beta_1$ in the model for the marginal mean of the outcome $E(Y_j) = \beta_0 + \beta_1 j$ ($j = 0, \ldots, 8$). The true values of $\beta_0$ and $\beta_1$ are 210 and $-2$, respectively.

There are 500 patients with 8 follow-up visits in addition to the baseline visit. Below are the first six rows of the data for the dropout process in the data.frame *dropoutdata*. 'sub' is subject ID; 'tall' is the period between visits $j-1$ and $j$ ($j = 1, \ldots, 8$); 'X1-X4' are baseline covariates; 'y' is the previous outcome $Y_{j-1}$; 'ycomp' is the current outcome $Y_j$; 'y_c' is the standardized previous outcome $Y^*_{j-1}$ ; 'ycomp_c' is the standardized current outcome $Y^*_j$; 'drop' is the indicator for dropout, which means that patient dropped out of the study between visits $j-1$ and $j$.

```
##   sub tall         X1          X2           X3         X4        y    ycomp
##     1    0  0.6743551 -0.06258534 -0.170461477  0.5470732 215.2877 218.3947
##     1    1  0.6743551 -0.06258534 -0.170461477  0.5470732 218.3947 225.3771
##     1    2  0.6743551 -0.06258534 -0.170461477  0.5470732 225.3771 226.0462
##     1    3  0.6743551 -0.06258534 -0.170461477  0.5470732 226.0462 210.0838
##     2    0 -0.9835526  0.08113915  0.008265763  0.9532584 200.3662 187.5812
##     3    0 -0.7895057  0.66668724 -0.796040418 -0.5971279 191.7273 182.2064
##          y_c      ycomp_c drop
##    0.1510764  0.239849486    0
##    0.2398495  0.439347096    0
##    0.4393471  0.458462068    0
##    0.4584621  0.002395352    1
##   -0.2752509 -0.640536692    1
##   -0.5220767 -0.794101455    0
```

These are the first few rows of the observed longitudinal data in the data.frame *simdata*. Note that the data.frame *dropoutdata* is prepared for fitting the dropout model to obtain the initial weights. The data.frame

*simdata* is prepared for fitting the regression model for the longitudinal outcome with weights to adjust for the selection bias due to dropout, where 'y' is the observed longitudinal outcome at visit numbers stored in the column 'tall', and 'y_c' is the standardized 'y'. It can be seen that the rows corresponding to 'drop=1' in the data.frame *dropoutdata* have been dropped in the data.frame *simdata*

```
##   sub tall         X1          X2           X3         X4        y        y_c
##     1    0  0.6743551 -0.06258534 -0.170461477  0.5470732 215.2877  0.1510764
##     1    1  0.6743551 -0.06258534 -0.170461477  0.5470732 218.3947  0.2398495
##     1    2  0.6743551 -0.06258534 -0.170461477  0.5470732 225.3771  0.4393471
##     1    3  0.6743551 -0.06258534 -0.170461477  0.5470732 226.0462  0.4584621
##     2    0 -0.9835526  0.08113915  0.008265763  0.9532584 200.3662 -0.2752509
##     3    0 -0.7895057  0.66668724 -0.796040418 -0.5971279 191.7273 -0.5220767
##     3    1 -0.7895057  0.66668724 -0.796040418 -0.5971279 182.2064 -0.7941015
```

## 2. Estimating the initial weights before calibration

**(1) Estimate $\boldsymbol{\alpha}$ with fixed selection function** To compute the initial weights before calibration, we estimate $\boldsymbol{\alpha}$, the coefficients of $X_1, X_2, X_3, X_4$ and the standardized previous outcome $Y^*_{j-1}$, while specifying the true selection function as $Y^*_j$.

$\boldsymbol{\alpha}$ is estimated by solving the estimating equation (4) in Su, Seaman and Yiu (2022) with the Newton-Raphson algorithm. *NRroot* is the function to implement the Newton-Raphson algorithm, which requires inputs for the initial values of the parameters *inipar*, and the function to be solved *infcn* and its first derivative *gradfunc*. In our case, the function to be solved is the left-hand side of the estimating equation (4) in Su, Seaman and Yiu (2022) and is named as *score*. Its first derivative is saved in *scoreder*. Below we set the initial values at zeros. Other choices can be the coefficient estimates of $X_1, X_2, X_3, X_4, Y^*_{j-1}$ in a standard logistic regression when no selection function is included.

```
alphahat<-NRroot(inipar=rep(0, 6), infcn=score, gradfunc=scoreder)
print(alphahat)
```

```
##             [,1]
## 1     1.95157990
## X1   -1.09688981
## X2    0.50897587
## X3   -0.29115111
## X4   -0.07535699
## y_c   0.26677992
```

Note that the functions *score* and *scoreder*, which are displayed below, both require inputs for the design matrix of the dropout model, the indicator of dropout, and also the sensitivity parameter value $\gamma$.

```
print(score)
```

```
## function (alpha = c(2, -1, 0.5, -0.25, -0.1, 0.2), gamma = 1,
##     x = dropoutdata[, c("X1", "X2", "X3", "X4", "y_c", "ycomp_c")],
##     ind = dropoutdata$drop)
## {
##     x <- cbind(1, x)
##     expx <- exp(-as.matrix(x) %*% as.matrix(c(alpha, gamma)))
##     obsx <- x[ind == 0, -(length(alpha) + 1)]
##     expxx <- (expx %*% t(as.matrix(rep(1, dim(x)[2] - 1))))
##     obsxx <- obsx * expxx[ind == 0, ]
##     missx <- -x[ind == 1, -(length(alpha) + 1)]
##     allscore <- rbind(obsxx, missx)
##     finalscore <- colSums(allscore)
##     as.matrix(finalscore)
```

```
## }
## <bytecode: 0x00000000162674e0>
```

```
print(scoreder)
```

```
## function (alpha = c(2, -1, 0.5, -0.25, -0.1, 0.2), gamma = 1,
##     x = dropoutdata[, c("X1", "X2", "X3", "X4", "y_c", "ycomp_c")],
##     ind = dropoutdata$drop)
## {
##     x <- cbind(1, x)
##     expx <- exp(-as.matrix(x) %*% as.matrix(c(alpha, gamma)))
##     obsx <- x[ind == 0, -c((length(alpha) + 1))]
##     expxx <- expx[ind == 0]
##     mat = 0
##     for (i in 1:length(expxx)) {
##         xvec <- obsx[i, ]
##         mat = mat - (t(as.matrix(xvec)) %*% as.matrix(xvec)) *
##             expxx[i]
##     }
##     mat
## }
## <bytecode: 0x0000000012aae360>
```

**(2) Calculate the initial weights** We use the function *newweight* to calculate the inverse of 1 minus the discrete-time hazard estimate and then take cumulative product of them. The results are the initial weights before calibration.

```
we_R<-newweight(alpha=alphahat)
weightsMLER<-ave(we_R,dropoutdata$sub,FUN=cumprod)
print(newweight)
```

```
## function (alpha = c(2, -1, 0.5, -0.25, -0.1, 0.2), gamma = 1,
##     x = dropoutdata[, c("X1", "X2", "X3", "X4", "y_c", "ycomp_c")])
## {
##     x <- cbind(1, x)
##     w = expit(as.matrix(x) %*% as.matrix(c(alpha, gamma)))
##     1/w
## }
```

### 3. Calibration

Before applying calibration to the initial weights, we create a column 'weights' to store the corresponding initial weights for the observed longitudinal data. Note that all baseline visits have a weight of one. Below are the first few rows of the observed longitudinal data with the initial weights.

```
##   sub tall         X1          X2           X3         X4        y        y_c
##     1    0  0.6743551 -0.06258534 -0.170461477  0.5470732 215.2877  0.1510764
##     1    1  0.6743551 -0.06258534 -0.170461477  0.5470732 218.3947  0.2398495
##     1    2  0.6743551 -0.06258534 -0.170461477  0.5470732 225.3771  0.4393471
##     1    3  0.6743551 -0.06258534 -0.170461477  0.5470732 226.0462  0.4584621
##     2    0 -0.9835526  0.08113915  0.008265763  0.9532584 200.3662 -0.2752509
##     3    0 -0.7895057  0.66668724 -0.796040418 -0.5971279 191.7273 -0.5220767
##     3    1 -0.7895057  0.66668724 -0.796040418 -0.5971279 182.2064 -0.7941015
##    weights
##   1.000000
##   1.230250
```

```
## 1.456853
## 1.706470
## 1.000000
## 1.000000
## 1.082061
```

As we are interested in regression coefficients in the model for the longitudinal outcome at each follow-up visit, we include interaction terms between the visit number $j$ (treated as a continuous variable) and $X_1, X_2, X_3, X_4, Y^*_{j-1}$ for calibration as well. The data for the final analysis is saved in the data.frame *simdatafinal*.

Below are the first few rows of the the data.frame *simdatafinal*. 'tX1', 'tX2', 'tX3', 'tX4', 'ty' are the interactions between the visit number $j$ and $X_1, X_2, X_3, X_4, Y^*_{j-1}$.

```
##  sub tall    ycomp        y         X1          X2           X3         X4 drop
##    1    0 218.3947 215.2877  0.6743551 -0.06258534 -0.170461477  0.5470732    0
##    1    1 225.3771 218.3947  0.6743551 -0.06258534 -0.170461477  0.5470732    0
##    1    2 226.0462 225.3771  0.6743551 -0.06258534 -0.170461477  0.5470732    0
##    1    3 210.0838 226.0462  0.6743551 -0.06258534 -0.170461477  0.5470732    0
##    2    0 187.5812 200.3662 -0.9835526  0.08113915  0.008265763  0.9532584    0
##    3    0 182.2064 191.7273 -0.7895057  0.66668724 -0.796040418 -0.5971279    0
##     droppi        y_c  weights       tX1         tX2        tX3       tX4
##  0.0000000  0.1510764 1.000000 0.0000000  0.00000000  0.0000000 0.0000000
##  0.1747507  0.2398495 1.230250 0.6743551 -0.06258534 -0.1704615 0.5470732
##  0.1455949  0.4393471 1.456853 1.3487101 -0.12517068 -0.3409230 1.0941465
##  0.1384061  0.4584621 1.706470 2.0230652 -0.18775601 -0.5113844 1.6412197
##  0.0000000 -0.2752509 1.000000 0.0000000  0.00000000  0.0000000 0.0000000
##  0.0000000 -0.5220767 1.000000 0.0000000  0.00000000  0.0000000 0.0000000
##         ty
##  0.0000000
##  0.2398495
##  0.8786942
##  1.3753862
##  0.0000000
##  0.0000000
```

We use the function *calibration* to implement the calibration procedure described in in Su, Seaman and Yiu (2022). This function requires the R package *nleqslv* and inputs for the longitudinal data with initial weights saved in a column called 'weights', a column 'sub' for the subject IDs, and a column 'tall' for the visit number (including the baseline visit). In addition, users need to provide the variable names for those variables to be included in the calibration restrictions. The calibrated weights are saved in a column 'Cweights' along with the longitudinal data provided.

```r
library(nleqslv)
simdatafinal<-calibration(simdatafinal,  var=c('tall', 'X1', 'X2', 'X3', 'X4', 'y_c',
                                                'tX1', 'tX2', 'tX3', 'tX4', 'ty'))
print(simdatafinal[1:6,], row.names=F)
```

```
##  sub tall    ycomp        y         X1          X2           X3         X4 drop
##    1    0 218.3947 215.2877  0.6743551 -0.06258534 -0.170461477  0.5470732    0
##    1    1 225.3771 218.3947  0.6743551 -0.06258534 -0.170461477  0.5470732    0
##    1    2 226.0462 225.3771  0.6743551 -0.06258534 -0.170461477  0.5470732    0
##    1    3 210.0838 226.0462  0.6743551 -0.06258534 -0.170461477  0.5470732    0
##    2    0 187.5812 200.3662 -0.9835526  0.08113915  0.008265763  0.9532584    0
##    3    0 182.2064 191.7273 -0.7895057  0.66668724 -0.796040418 -0.5971279    0
##     droppi        y_c  weights       tX1         tX2        tX3       tX4
##  0.0000000  0.1510764 1.000000 0.0000000  0.00000000  0.0000000 0.0000000
```

```
##  0.1747507  0.2398495 1.230250 0.6743551 -0.06258534 -0.1704615 0.5470732
##  0.1455949  0.4393471 1.456853 1.3487101 -0.12517068 -0.3409230 1.0941465
##  0.1384061  0.4584621 1.706470 2.0230652 -0.18775601 -0.5113844 1.6412197
##  0.0000000 -0.2752509 1.000000 0.0000000  0.00000000  0.0000000 0.0000000
##  0.0000000 -0.5220767 1.000000 0.0000000  0.00000000  0.0000000 0.0000000
##         ty Cweights
##  0.0000000 1.000000
##  0.2398495 1.386438
##  0.8786942 1.624677
##  1.3753862 1.853933
##  0.0000000 1.000000
##  0.0000000 1.000000
```

Below are the violin plots and scatter plot of the initial weights and calibrated weights.

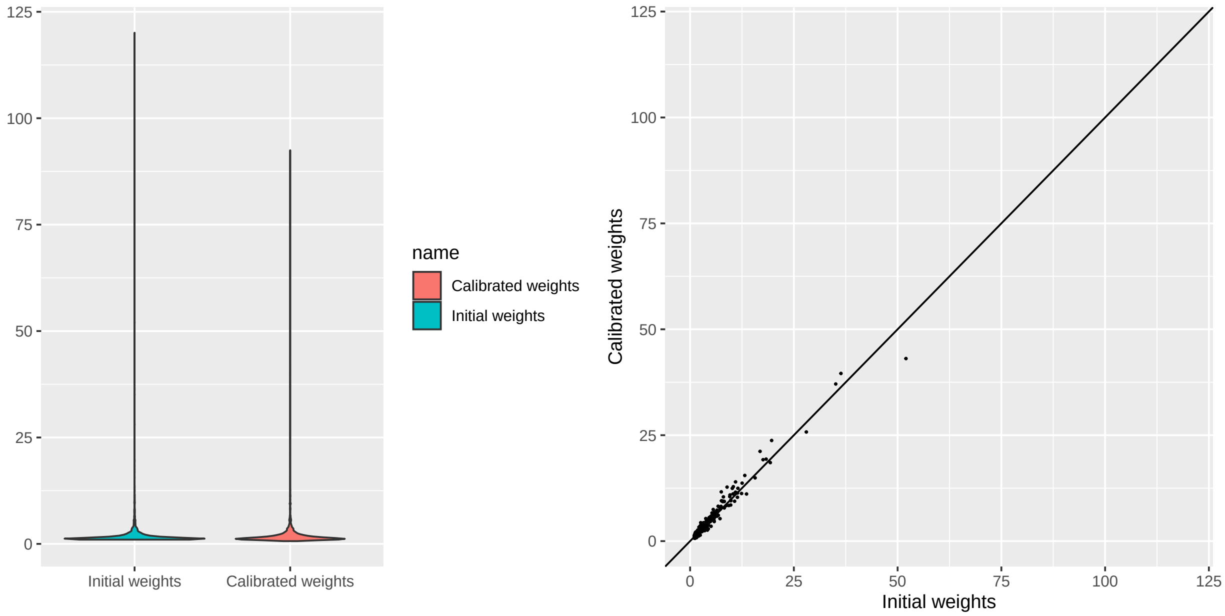


### 4. Implement calibrated IPCWEs

It is straightforward to implement calibrated IPCWEs using the function *lm* with the calibrated weights, as well as the initial weights without calibration.

```
IPCWE<-lm(y~tall,data=simdatafinal,weights=weights)
CIPCWE<-lm(y~tall,data=simdatafinal,weights=Cweights)
summary(IPCWE)
```

```
##
## Call:
## lm(formula = y ~ tall, data = simdatafinal, weights = weights)
##
## Weighted Residuals:
##     Min      1Q  Median      3Q     Max
## -708.55  -19.17   10.49   39.14  150.53
##
## Coefficients:
##             Estimate Std. Error t value Pr(>|t|)
## (Intercept) 210.4802     1.4344 146.739   <2e-16 ***
```

```
## tall         -2.4510     0.2945  -8.322   <2e-16 ***
## ---
## Signif. codes:  0 '***' 0.001 '**' 0.01 '*' 0.05 '.' 0.1 ' ' 1
##
## Residual standard error: 52.08 on 2472 degrees of freedom
## Multiple R-squared:  0.02725,    Adjusted R-squared:  0.02686
## F-statistic: 69.25 on 1 and 2472 DF,  p-value: < 2.2e-16
```

```
summary(CIPCWE)
```

```
##
## Call:
## lm(formula = y ~ tall, data = simdatafinal, weights = Cweights)
##
## Weighted Residuals:
##     Min      1Q  Median      3Q     Max
## -643.76  -19.18   10.34   39.12  142.94
##
## Coefficients:
##             Estimate Std. Error t value Pr(>|t|)
## (Intercept) 209.1986     1.4099 148.381  < 2e-16 ***
## tall         -2.0052     0.2942  -6.817 1.17e-11 ***
## ---
## Signif. codes:  0 '***' 0.001 '**' 0.01 '*' 0.05 '.' 0.1 ' ' 1
##
## Residual standard error: 52.11 on 2472 degrees of freedom
## Multiple R-squared:  0.01845,    Adjusted R-squared:  0.01805
## F-statistic: 46.47 on 1 and 2472 DF,  p-value: 1.165e-11
```

### 5. Confidence intervals

To construct confidence intervals based on Sandwich variance estimator, we use the *geeglm* function from the R package *geepack*.

```
library(geepack)
geeMLE<-geeglm(y~tall, data=simdatafinal, weights=weights, id=sub,  corstr = 'independence')
LL_MLE=coef(geeMLE)-1.96*coef(summary(geeMLE))[2]
UL_MLE=coef(geeMLE)+1.96*coef(summary(geeMLE))[2]

geeCMLES<-geeglm(y~tall, data=simdatafinal, weights=Cweights, id=sub,  corstr = 'independence')
LL_CMLES=coef(geeCMLES)-1.96*coef(summary(geeCMLES))[2]
UL_CMLES=coef(geeCMLES)+1.96*coef(summary(geeCMLES))[2]
```

The 95% confidence interval based on Sandwich variance estimator and initial MLE weights for $\beta_0$ and $\beta_1$ are [204.15, 216.81], [-5.17, 0.27], respectively.

The 95% confidence interval based on Sandwich variance estimator and calibrated weights for $\beta_0$ and $\beta_1$ are [203.39, 215.01], [-4.34, 0.33], respectively.

For bootstrap/jackknife confidence intervals, the initial weights from the original data can be used and then calibration can be done using the *calibration* function in the bootstrap/jackknife samples. Or initial weights can be recalculated for each bootstrap sample.
